# Supplementary material for: Exploring Gut Microenvironment in Colorectal Patient with Dual-Omics Platform: A Comparison with Adenomatous Polyp or Occult Blood
Source: Biomedicines. 2022 Jul 19;10(7):1741. doi: 10.3390/biomedicines10071741 (PMC9313112; doi:10.3390/biomedicines10071741)
Supplement: Supplementary file 1 [file biomedicines-10-01741-s001.zip › Supplemental Table S3.pdf]

Supplementary Table S3

POS Mode

CpdName	KEGGID	Microbe	Hsa	CRC	Healthy	Fold change (log2)	VIP	*p* value
Mirtazapine	C07570	NA	NA	4.98E-06	4.22E-05	-3.08	2.384	0.074217
Quercitrin	C01750	NA	NA	2.61E-05	0.000199	-2.93	2.131	0.028223
Xanthoxylin	C10726	NA	NA	1.27E-05	1.82E-06	-2.84	1.983	0.017969
Salicylic acid	C00805	1	1	4.66E-06	2.54E-05	-2.44	1.846	0.005715
Fasciculic acid B		NA	NA	5.44E-05	0.000216	-1.98	1.906	0.029946
Capsaicin	C06866	1	1	1.95E-05	6.78E-05	-1.79	1.848	0.047629
Tricetin	C10192	NA	NA	0.019236	0.059918	-1.63	1.732	0.020291
2,5-Dimethylpyrazine		NA	NA	1.07E-05	2.96E-05	-1.46	1.667	0.024715
Glycyrrhizin	C02284	NA	NA	1.39E-05	3.83E-05	-1.45	1.545	0.023744
Gabapentin	C07018	NA	NA	2.29E-05	5.39E-05	-1.23	1.603	0.037146
Adenine	C00147	1	1	4.2E-05	9.74E-05	-1.21	1.621	0.029069
Metoprolol	C07202	NA	NA	8.47E-05	0.000189	-1.15	1.515	0.048418
Thymol	C09908	NA	NA	0.000139	0.000297	-1.09	1.609	0.031962
Ritalinic acid		NA	NA	8.99E-06	1.83E-05	-1.02	1.608	0.010903
N1-Acetylspermine	C02567	NA	NA	4.63E-05	9.28E-05	-1.0029	1.507	0.031913
L-Carnitine	C00318	NA	1	1.23E-06	3E-07	2.03	1.841	0.036663
Methsuximide		NA	NA	9.84E-06	2.38E-06	2.07	1.513	0.005043
L-Phenylalanine	C00079	1	1	0.0002	4.54E-05	2.13	1.674	0.012917
Ofloxacin	C07321	NA	NA	2.06E-05	4.08E-06	2.33	1.547	0.039726
Cilastatin	C01675	NA	NA	2.05E-05	3.94E-06	2.37	1.861	0.015044
N-Desmethylvenlafaxine		NA	NA	7.91E-06	1.2E-06	2.72	1.775	0.021136
Lovastatin	C07074	NA	NA	3.84E-06	5.29E-07	2.861	1.893	0.026004
Urobilinogen	C05791	1	1	3.37E-05	4.56E-06	2.886	1.664	0.043053
Sorbitan palmitate		NA	NA	0.000112	1.37E-05	3.029	2.045	0.025181
Acetaminophen	C06804	NA	1	4.87E-05	5.92E-06	3.041	2.015	0.042645
L-Valine	C00183	1	1	2.33E-05	2.77E-06	3.071	2.137	0.015123
Dehydroepiandrosterone	C01227	1	1	0.000327	3.26E-05	3.325	2.253	0.027779
Gibberellin A3	C01699	1	NA	0.000388	2.53E-05	15.359	2.941	0.003978
N,N-Dimethylaniline	C02846	NA	NA	6.74E-05	4.33E-06	15.579	2.961	0.037457
S-Adenosylhomocysteine	C00021	1	1	0.0009	1.12E-06	40.404	2.644	0.029642

NEG Mode

CpdName	KEGGID	Microbe	Hsa	CRC	Healthy	Fold change (log2)	VIP	*p* value
Niflumic Acid	C13698	NA	NA	3.03E-06	0.0001	-5.05	2.038	0.035863
Fumonisin A2		NA	NA	2.92E-05	0.000214	-2.87	1.886	0.053838
(S)-4',5,7-Trihydroxy-6-prenylflavanone	C09832	NA	NA	0.000226	0.001014	-2.16	1.832	0.085713
Glucobrassicin		NA	NA	3.72E-05	0.000135	-1.85	1.849	0.001381
Tricosanoic acid		NA	NA	0.000715	0.002279	-1.67	1.692	0.032453
4-Dodecylbenzenesulfonic Acid		NA	NA	0.000224	0.000591	-1.42	1.54	0.019116
L-Tryptophan	C00078	1	1	0.00044	0.000969	-1.14	1.607	0.004386
Creatine	C00300	1	1	0.000806	0.001588	-0.97	1.577	0.031535
3-Methoxy-4-Hydroxyphenylglycol sulfate		NA	NA	0.002236	0.001098	1.03	1.612	0.053077
5-HETE	C04805	1	1	1.05E-05	4.76E-06	1.13	1.647	0.053801
Atorvastatin	C06834	NA	NA	0.000213	7.8E-05	1.45	1.811	0.036553
Pentadecanoic acid	C16537	NA	NA	0.008059	0.001952	2.04	2.057	0.025142
Estrone glucuronide	C11133	NA	1	2.85E-05	6.89E-06	2.047	2.124	0.045259
Stearic acid	C01530	1	1	0.000354	7.86E-05	2.17	2.232	0.017697
Allocholic acid	C17737	1	NA	0.004191	5.39E-05	6.28	2.247	0.038343
