# Supplementary figures and images for: Exploring Gut Microenvironment in Colorectal Patient with Dual-Omics Platform: A Comparison with Adenomatous Polyp or Occult Blood
Source: Biomedicines. 2022 Jul 19;10(7):1741. doi: 10.3390/biomedicines10071741 (PMC9313112; doi:10.3390/biomedicines10071741)

Supplementary Figure S1

A

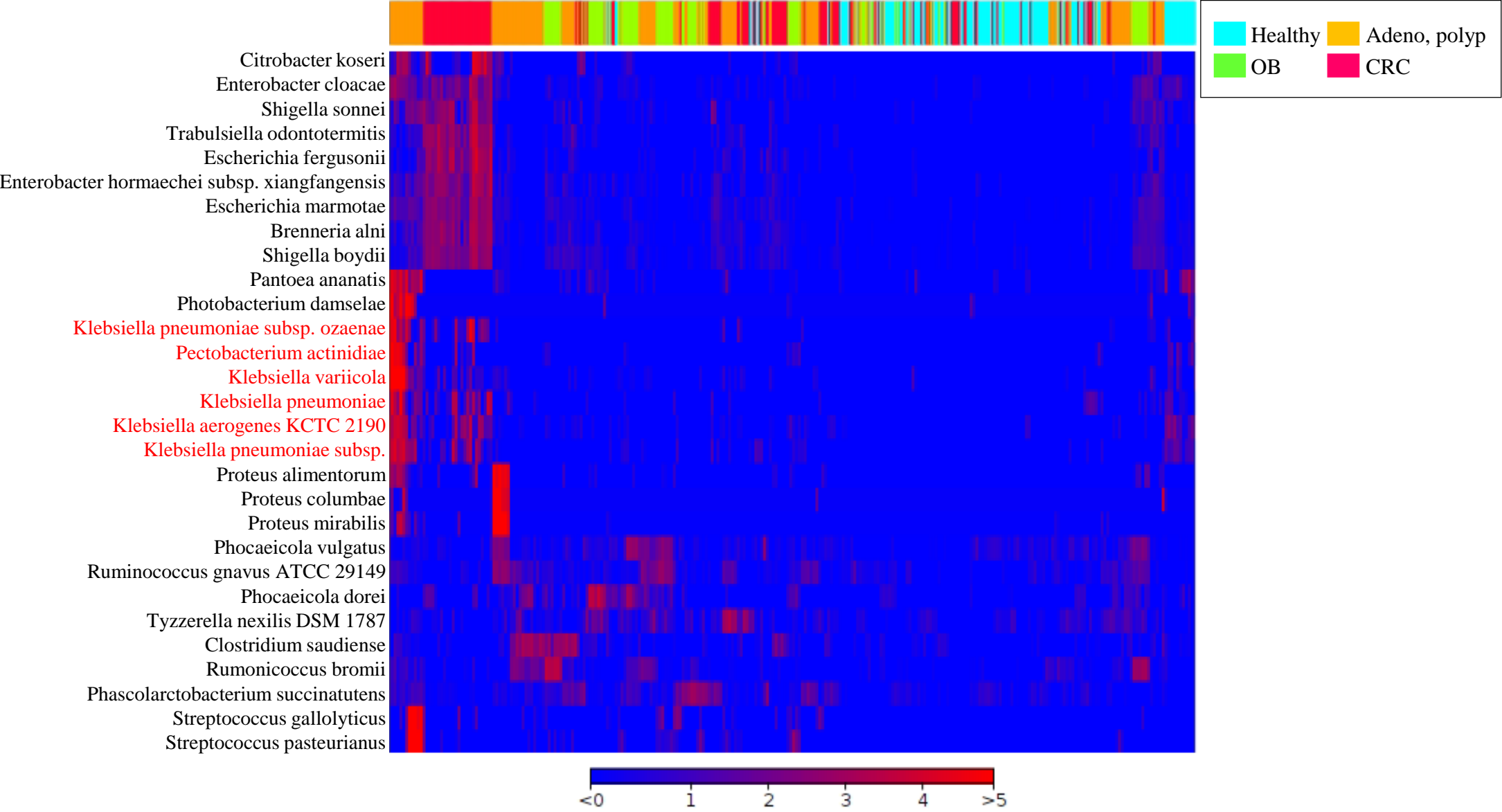

B

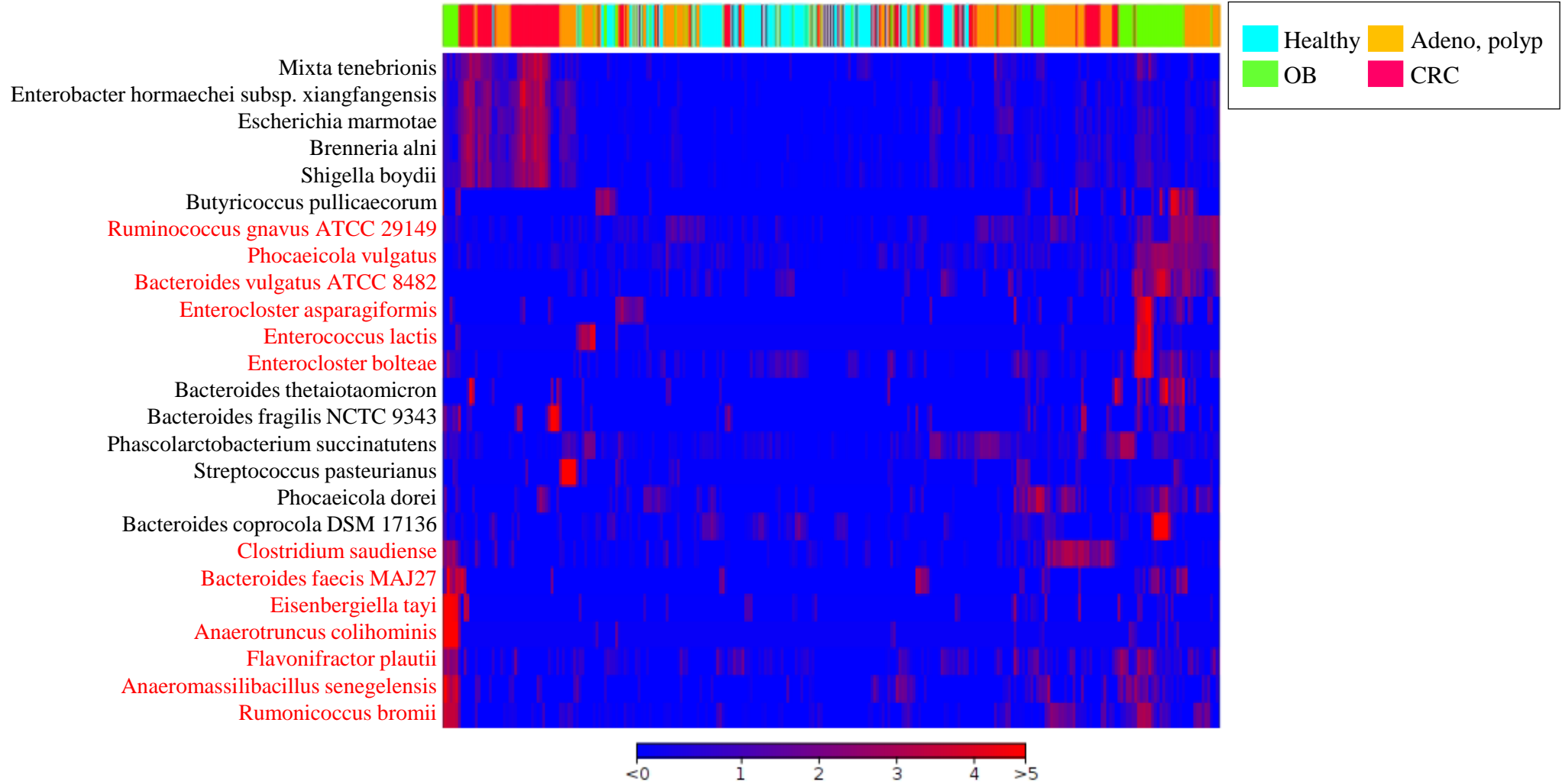

Supplement: Supplementary file 1 [file biomedicines-10-01741-s001.zip › Supplemental Figure S1.pdf]
